# 

**Published:** 1889-05-18

**Authors:** 


### NOTICE TO CORRESPONDENTS.

All MS., Letters, Books for Review, and other matters intended for the Editor, should be addressed The Editor, The Lodge, Porchester Square, London, W.

### Notice to Advertisers and Subscribers.

All Advertisements, Orders for Copies of The Hospital, and Business Communications should be addressed to The Publisher, *not to the Editor* The Hospital, 139-140, Salisbury-court, Fleet-street, E.C.

Vol. V., now ready, 4s., post free. Cases for binding, may be had of the Publisher, at 1s. 6d. each.

☞ Subscribers to the Hospital Annual are requested to send post-card to the Publisher if they have not received their copies, it having been discovered that a few copies have been delayed by carriers.

### To Residents, Secretaries, and Matrons.

The Editor will be glad to have the Regulations and each Report as issued by Asylums, Hospitals, Convalescent and Nursing Institutions, as well as those of other Charities, and an early copy in duplicate of all Appeals and Festival Notices, etc., addressed to *The Editor, The Lodge, Porchester-square, London, W.* Any superintendent, secretary, matron, or other chief official who regularly sends such information may be registered, on application to the Publisher, to receive free of cost a cover for binding The Hospital in half-yearly volumes.

### Amusements and Relaxation.

### Word Competition.

Commenced April 6th, Ends June 29th.

Three Prizes of 15s., 10s., 5s., will be given for the largest number of words derived from the words set for dissection.

Proper names, abbreviations, foreign words, words of less than four letters, and repetitions are barred; plurals, and past and present participles of verbs, are allowed. Nuttall's *Standard* dictionary only to be used.

N.B.—Word dissections must be sent in WEEKLY, arranged alphabetically, with correct total affixed.

The word for dissection for this, the *Seventh* week of the quarter, being

"LEIGHTON."

### Results of Fifth Week.

| Names. | May 9th. | Totals. | Names. | May 9th. | Totals. |
|---|---|---|---|---|---|
| Patience | 115 | 502 | Tiny | 101 | 413 |
| Nurse Duty | 100 | 480 | N'importe Qui | 90 | 389 |
| Lightowlers | 104 | 480 | Pollie | 71 | 361 |
| Scrooge | 93 | 461 | W. G. R. | 90 | 360 |
| Reldas | — | 250 | Robe | — | 150 |
| Tinie | 115 | 505 | Beryl | 83 | 338 |
| K. G. | 101 | 463 | Nesta | — | 124 |
| Broxbourne | 101 | 469 | Daffodil | — | 89 |
| Lauriston | 104 | 472 | A Schoolgirl | — | 130 |
| Spes | 99 | 468 | Montague | — | 79 |
| Gretta | — | 224 | Sheeny | — | 111 |
| Francesca | 95 | 459 | Alice R | — | 63 |
| Heatherbell | — | 185 | Belfast | — | 163 |
| Sycamore | — | 201 | Gentian | — | 209 |
| Arctus | 75 | 406 | Hope | — | 39 |
| Amicus | 113 | 468 | Louie | — | 139 |
| Scratcher | — | 197 | Hamilton | — | 121 |
| Jemima | — | 138 | E. M. | — | 120 |
| Concours | 96 | 410 | F. J. D. | — | 112 |
| C. J. S. | — | 134 | Ladyr | — | 102 |
| See-Saw | 96 | 427 | Surgical Nurse | — | — |
| Rosemont | — | 173 | Violet | — | 95 |
| Jenny Wren | 103 | 245 | L. Pearce | — | 32 |
| Eric | 92 | 402 | Contentio | 81 | 81 |

### Notice to all Correspondents.

N.B.—All letters referring to this page which do not arrive at 139 and 140, Salisbury-court, London, E.C., by the *first post on Thursdays*, and are not addressed PRIZE EDITOR, will in future be disqualified and disregarded.

### Conditions.

I. Every paper sent in must be clearly written, and one side only must be used. All competitors must mark at the head of their papers the number of their competition.

II. Each paper must be signed by the author with his or her real name and address. A *nom de plume* may be added if the writer does not desire to be referred to by us by his real name. In the case of all prize-winners, however, the real name and address will be published.

III. All communications relating to prize competitions should be addressed to "The Prize Editor, The Hospital, 139 and 140, Salisbury-court, London, E.C." Competitors are requested not to include in letters, etc., to the Prize Editor communications on matters of other business. All letters must be fully prepaid.

IV. The Prize Editor of The Hospital is the sole judge of the competitions, and his decision is in all cases final and without appeal.

V. The Prize Editor reserves the right to publish any paper sent in, whether it receives a prize or not. He does not hold himself responsible for any MSS. sent, nor can he undertake to return rejected competitions.

### Scraps and Gleanings.

Horton Infirmary has a balance of £334 at the bank.

Sir William Jenner has been staying at Windsor Castle.

Mr. Clifford will shortly publish a life of Father Damien.

The death is announced of Dr. McDonnell, a well-known physician of Dublin.

Mrs. Ernest Hart has secured a large contract of work for her poor of Donegal.

The Society of Apothecaries will hold their next examination in arts on June 7 and 8.

Mr. Walter Besant witnessed two gymnastic displays at the People's Palace last week.

The bazaar at Bournemouth brought in over £2,000 for the Royal Victoria Hospital.

The Princess Louise will open the Children's Hospital, Leicester, on Friday, the 31st inst.

Dr. Edward Liveing has been appointed registrar to the Royal College of Physicians.

The Monsall Board refuses to undertake the charge of all the fever patients from Salford.

Mr. Bartholomew has given the old Court House, Goole, to be made into a cottage hospital.

The Surgical Appliance Society held their annual dinner at the Hotel Metropole last Monday.

Dr. W. Hunter will lecture on "Transfusion" at the Royal College of Surgeons on June 24, 26, and 28.

The medical profession of Rio de Janeiro have adopted Dr. Domingos Freir's system of inoculation against yellow fever.

The Prince of Wales will unveil the statue of the Queen at the Surgeons' Examination Hall, Embankment, on May 24.

Dr. Walter Verdon (M.D. Brussels) has started a movement to establish a children's hospital in the vicinity of Stockwell.

The Queen has forwarded a donation of fifty guineas to the Royal Maternity Charity, Finsbury-square, of which she is patron.

The 57th annual report of Barrington's Hospital, Limerick, tells of sunshine after rain. There is a balance in the bank of £127.

The Mansion House Council on the Housing of the Poor have turned their attention to Woolwich, where things are in a shocking condition.

Signs of the Times.—Not one of the gentlemen arrested during the late raid upon betting clubs gave his profession as "medical student."

Mr. H. Murray, Mr. G. O. Richard, Mr. R. W. Rouw, Mr. J. Mansbridge, and Mr. W. A. Maggs, have been appointed dental surgeons at Guys.

The Committee of the Convalescent Home for Friendly Societies in the North of England are pushing forward their scheme, which is highly approved.

The medical officer of health for Ventnor reports that the death-rate there is the lowest in England, being 5·7 per 1,000 for 1888, and only 8·3 per 1,000 for 1887.

The German National Exhibition of Appliances for the Prevention of Accidents has been opened in the Prussian Exhibition Palace, by the Emperor of Germany.

It is proposed to build a small fever hospital at Portree, Skye, as typhoid, typhus, and famine fever (and this is Christian England!) are common on the island.

The furnishing of the Hartley Memorial Wing to Sunderland Infirmary has been carried out with wondrous rapidity, thanks to the generosity of many private individuals.

Mr. G. W. Hastings, M.P., has accepted the post of president of the Congress of the Sanitary Institute, which is to be held in Worcester, commencing September 24th.

There is still some enthusiasm in Ireland. The Mayor of Cork, speaking of the report of the North Infirmary, called it "a beautiful account of a hard year's work."

Those London firemen who have distinguished themselves in the discharge of their perilous duties are to receive medals, and they are to be presented by the Princess of Wales.

The Duke of Norfolk last week opened a new annexe to the Eastern Counties Asylum at Colchester. He was accompanied by the Marquis of Bristol, Lord and Lady Brooke, etc.

Dr. Barry's report on the smallpox epidemic points out that the Borough Hospital, Sheffield, acted as a distributor of the disease. We hope this hospital will shortly be swept away.

The Mansion House Fund for the relief of the sufferers by the famine in China is now closed. The subscriptions amounted in the aggregate to over £30,000, raised in all parts of the kingdom.

We noticed with admiration the advertisements inserted in the Guide to the Grimsby Bazaar; they must have paid the cost of printing the guide. How excellently thrifty are our friends in the North!

The King of the Belgians has promised the handsome contribution of £500 towards that portion of the Durham College of Science at Newcastle-on-Tyne to be erected to perpetuate the memory of George and Robert Stephenson.

The festival dinner of the National Hospital for the Paralysed and Epileptic will be held on Thursday, May 23rd, at the Whitehall rooms of the Hotel Metropole, when His Grace the Lord Archbishop of Canterbury will take the chair.

The terrible outbreak of measles which raged with such virulence throughout the Staffordshire potteries some three months ago is now of a more alarming character than ever. No fewer than 250 cases are reported in Hanley alone.

As Dr. Cromie, medical officer for the Clough district of the county of Down, was returning from visiting a patient at Wood Grange, he was overtaken by a thunderstorm, and the car in which he was riding was struck by lightning. The driver was killed on the spot, but Dr. Cromie escaped untouched.

